# From theory to research: Interpretational guidelines, statistical guidance, and a shiny app for the model of excellencism and perfectionism

**DOI:** 10.1177/08902070231221478

**Published:** 2023-12-20

**Authors:** Patrick Gaudreau, Benjamin JI Schellenberg, Matthew Quesnel

**Affiliations:** 1 School of Psychology, 151181 University of Ottawa, Ottawa, ON, Canada; 2 Faculty of Kinesiology and Recreation Management, 8664 University of Manitoba, Winnipeg, MB, Canada; 3 8664 University of Manitoba, Winnipeg, MB, Canada

**Keywords:** savoring, enjoyment, dropout intention, sport performance, moderator

## Abstract

After decades of research and debates about whether perfectionism is healthy or unhealthy, the Model of Excellencism and Perfectionism (MEP) recently differentiated between people striving for high standards (excellence strivers) and those pursuing perfectionistic standards (perfection strivers). In this study, we devised and tested an interpretational framework of nine scenarios to help determine whether perfectionism is beneficial, unneeded, or harmful by comparing the outcomes of excellence and perfection strivers. In a cross-sectional study with university students (*N* = 271; Study 1), we found that perfection strivers savor positive school events less and have greater dropout intentions than excellence strivers. In a prospective/longitudinal design with college-aged athletes (*N* = 296; Study 2), perfectionism was associated with higher athletic achievement. However, perfection strivers who failed to attain their goals experienced lower savoring and enjoyment than excellence strivers. Our findings highlighted the value of our interpretational scenarios as a hub to facilitate the comparison of MEP findings, while showing how to test MEP hypotheses with five popular statistical analyses. Furthermore, the MEP Shiny App is a valuable contribution to expedite the process of comparing the outcomes of excellence and perfection strivers. Overall, this research forged a substantive-methodological pathway that strengthens and enhances the practicality of the MEP.

## Plain language summary

Many people aspire to achieve excellence without ever trying to attain perfection. Excellence and perfection are different endpoints that set the tone for distinct aiming, striving, phenomenological experiences, and outcomes. A recent theory introduced the concept of excellencism in order to differentiate individuals striving for high yet flexible standards (referred to as excellence strivers) from those who relentlessly and inflexibly chase perfection (known as perfection strivers). In the current research, our overarching goal was to enhance theory with a new framework that would make it easier for researchers to use the Model of Excellencism and Perfectionism (MEP). To achieve our goal, we conducted two studies with cross-sectional and longitudinal methods with university students (Study 1, *N* = 271) and college-aged athletes (Study 2, *N* = 296). We created the online MEP Shiny App (https://model-of-excellencism-and-perfectionism.shinyapps.io/Shiny_Version2/) to simplify the process of testing and interpreting the differences between excellence and perfection strivers. Our findings underscored the importance of separating excellencism and perfectionism. In Study 1, perfection strivers savored positive school events less and had greater dropout intentions than excellence strivers. In Study 2, perfectionism was associated with higher goal attainment in sports. However, perfection strivers who failed to attain their goals experienced less savoring and enjoyment than excellence strivers. The positive sports experiences of perfection strivers depended on whether or not they attained their goals. Perfectionism is tricky and can be interpreted as a double-edged sword. It can be seen as beneficial, unneeded, or harmful depending on which outcomes and contexts researchers are looking at. Our findings highlighted the value of our newly proposed interpretational scenarios to make the MEP more accessible to other researchers and practitioners.

## Introduction

Jean and Jordan are often described by their friends as highly motivated and goal-driven individuals. An important goal in their lives is to be competent, very successful, and to produce high-quality work. They both aim and strive toward very high standards. Jordan, however, aims and strives toward something far more elusive and unattainable than Jean. Jordan not only wants to be a competent person; Jordan wants to be a perfect person. Although Jean wants to perform very well and works hard until attaining excellence, Jordan wants to perform perfectly and works relentlessly in an excessive attempt to reach unattainable ideals of perfection. As a result, Jean is generally satisfied with their performance and goal success, whereas Jordan remains relatively unsatisfied and often feels like a failure. It seems like nothing is ever good enough for Jordan who frequently lives with concerns over mistakes, doubts about their actions, self-criticism, and a sense of social pressure and obligation to be perfect.

Over the past three decades, the distinct psychological experiences of people like Jean and Jordan remained indistinguishable in a majority of the theoretical and empirical work on perfectionism. Recently, Gaudreau (2019) put forth the Model of Excellencism and Perfectionism (MEP) to formally differentiate the *excellencism* of Jean and the *perfectionism* of Jordan. Excellencism represents a “tendency to aim and strive toward very high yet attainable standards in an effortful, engaged, and determined yet flexible manner” (Gaudreau, 2019, p. 200). It needs to be contrasted with perfectionism, which represents “a tendency to aim and strive toward idealized, flawless, and excessively high standards in a relentless manner” (Gaudreau, 2019, p. 200). According to the MEP, excellencism and perfectionism are similar yet different. They are *similar* because they both involve aiming and striving toward very high standards. They are *different* because they involve distinct endpoints and ways of striving and they are accompanied by unique phenomenological experiences. As such, excellencism differs from perfectionism because it operates without the perfectionistic concerns (e.g., concerns over mistakes, doubts about actions, contingent self-worth, and perceived social pressure) that characterize the psychological experience of being a person who pursues perfection (e.g., Gaudreau, 2021; Gaudreau et al., 2022).

Many scholars have anecdotally talked about some of these differences (e.g., Adderholdt & Goldberg, 1999; Shafran et al., 2010). Before the MEP, however, limited studies differentiated the high standards involved in excellencism from the perfectionistic standards involved in perfectionism (Blasberg et al., 2016; Osenk et al., 2020). Conflating both standards into a single measure created a strawman fallacy that potentially overestimated the benefits and underestimated the risks associated with perfectionistic standards (e.g., Gaudreau, 2019; Gaudreau et al., 2023). As noted by Gaudreau (2019), this “conceptual gap has been, thus far, the Achilles heel of the perfectionism literature” (p. 210). The conceptual separation proposed and operationalized in the MEP is a useful advance to accurately estimate the specific benefits and risks associated with excellencism and perfectionism. The MEP now offers the needed conceptual and theoretical foundations to strengthen our inferences about the psychological outcomes of people who pursue excellence (Jean) and people who pursue perfection (Jordan).

A growing stream of research already emerged to investigate the novel assertions from the MEP (e.g., Cheek & Goebel, 2020; Gaudreau et al., 2021; Gaudreau et al., 2022; Gaudreau & Schellenberg, 2022; Goulet-Pelletier et al., 2022; Grieve et al., 2022). What is still missing is a clearer roadmap to help researchers transform the theory into actionable research. In this study, our goal was to create a substantive-methodological synergy between the hypotheses of the MEP and the typical statistical analyses used by researchers in the extant perfectionism literature. First, we introduced *nine different interpretational scenarios* that will help researchers draw theory-driven inferences from distinct patterns of excellencism and perfectionism effects. Second, we reported the results of two studies with five empirical examples—each using a different variable-centered statistical analysis. Our analyses are accompanied by our newly developed Shiny App designed to facilitate the process of using the effects of excellencism and perfectionism to compare the outcomes of excellence strivers, perfection strivers, and people who pursue lower standards (i.e., nonexcellence/nonperfection strivers).

### The model of excellencism and perfectionism

Over the last thirty years, perfectionism has been conceptualized and studied as a multidimensional personality disposition (e.g., Frost et al., 1990; Hewitt et al., 2017; Slaney et al., 2001). As reiterated in a recent review (Gaudreau et al., 2023), the MEP continues and extends this research tradition with two original propositions: (a) the distinction between the core definitional feature of perfectionism and the signature expressions of perfectionism (Gaudreau, 2021) and (b) the difference between excellencism and perfectionism (Gaudreau, 2019).

As reviewed by Gaudreau (2021), many multidimensional definitions of perfectionism assumed that perfectionistic standards *are accompanied by* perfectionistic concerns (e.g., Frost et al., 1990; Sirois & Molnar, 2016; Stoeber, 2018). To improve consistency between definitions and measurement, the MEP proposes a new distinction between the core definitional feature and the signature expressions of perfectionism (e.g., Gaudreau, 2021; Gaudreau et al., 2023). On the one hand, the *core definitional feature of perfectionism* is represented by the perfectionistic standards needed to separate perfectionism from excellencism. “Perfectionism starts where excellencism ends” and beyond excellence, the aiming and striving toward perfection creates a set of phenomenological experiences that characterize the unique cognitive, behavioral, and social experiences of being a person who pursues perfection (Gaudreau et al., 2023, p. 381). Characteristics such as evaluative concerns, doubts about actions, socially prescribed perfectionism, automatic thoughts about perfection, and perfectionistic self-presentation are reengineered as *signature expressions of perfectionism* because they come with perfectionism but not with excellencism (see Gaudreau, 2021 for a detailed list of signature expressions). They are repositioned outside the core definitional feature to give them their own crucial space in the conceptual landscape of perfectionism (for a complete description of the role of signature expressions in the MEP, see Gaudreau et al., 2023, pp. 382–387). Perfectionistic standards can be studied on their own as the core definitional feature of perfectionism, but they sure come with many signature expressions that play a pivotal role in the development and maintenance of psychological adjustment and maladjustment. In the current study, we focused on the core definitional feature of perfectionism to determine whether perfectionism relates to beneficial, unneeded, or harmful outcomes compared to those associated with excellencism.

The English language contains many words and expressions to characterize the meanings of excellence and perfection (Gaudreau, 2019, see p. 200). People who pursue excellence like Jean are likely to use words such as competent, very good, productive, skillful, capable, and successful to define their goals and standards. People who pursue perfection like Jordan are likely to also recognize themselves in these adjectives. In that sense, excellencism and perfectionism have some conceptual similarities. However, additional words exist to capture the unique ingredients that differentiate perfectionism from excellencism. Those who pursue perfection like Jordan are likely to be described as aiming and striving toward something more extreme, exact, flawless, impeccable, and error-free. These additional characteristics—the ones that are unique to perfectionism—form the core definitional feature that differentiates perfectionism from excellencism (e.g., Gaudreau, 2021).

**Figure 1.**
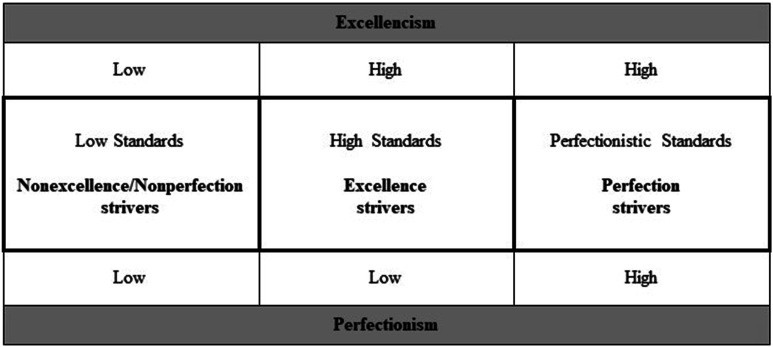
Operational definitions based on the dimensions of excellencism and perfectionism. Note. Low: One standard deviation below the mean; High: One standard deviation above the mean.

Excellencism and perfectionism are distinct but related dimensions sharing a partially conjunctive relation (see Gaudreau, 2019, Figure 1, p. 200). This has four interrelated implications. First, excellencism and perfectionism are conceptually distinguishable but correlated. Indicators used to measure excellencism and perfectionism occupy their own conceptual space in an oblique two-factor framework. Both exploratory and confirmatory factor analyses have offered support for their conceptual distinctiveness (Gaudreau et al., 2022). Second, inferences can be made about the outcomes of individuals with low standards, high standards, and perfectionistic standards based on scores of excellencism and perfectionism (see Figure 1).^1^ Excellence strivers like Jean are not pursuing perfection. They have high excellencism coupled with low perfectionism. In contrast, perfection strivers like Jordan inescapably pursue excellence during their quest toward perfection. Therefore, they have high excellencism and high perfectionism. Third, to evaluate the effects of perfectionism, one has to understand that perfection strivers aim and strive beyond the high standards involved in excellencism. Perfectionism is an extreme case that extends beyond the pursuit of excellence. Excellencism rather than non-perfectionism (i.e., nonexcellence/nonperfection strivers) is therefore the needed comparison point to properly estimate whether perfectionism is beneficial, unneeded, or harmful (Gaudreau, 2019; Gaudreau et al., 2022). Based on a behavioral economy analogy (e.g., Brue, 1993), the MEP proposes that investing more resources into something to extract the exact same amount of returns is inefficient, cost ineffective, and unneeded (see Gaudreau, Figure 3, p. 203). Therefore, showing incremental benefits of perfectionism over and above excellencism would be needed to conclude that perfectionism is beneficial. Fourth, perfectionism and excellencism are related, but the pursuit of perfection goes above and beyond the pursuit of excellence. Therefore, bivariate correlations are insufficient to properly estimate the associations of perfectionism with outcomes (see Gaudreau et al., 2023, p. 394). Both excellencism and perfectionism should be measured and mutually controlled for using a multivariable statistical analysis in order to estimate their unique/main effects. Estimates of the unique/main effects of excellencism and perfectionism are then used to calculate and compare the predicted values of the outcome (i.e., dependent variable) associated with nonexcellence/nonperfection strivers, excellence strivers, and perfection strivers (see Gaudreau, 2019, pp. 205–207).

Different variable-centered statistical analyses (e.g., multiple regression) can be applied depending on the research question, design, and nature of the dependent variables. For example, multiple regression (see Study 2 in Gaudreau et al., 2022) and multivariate multiple regression (see Goulet-Pelletier et al., 2022) were used in MEP research to examine one or many dependent variables assumed to have a continuous distribution. In contrast, logistic regression was used to examine a dichotomous dependent variable (see Study 4 in Gaudreau et al., 2022), whereas zero-inflated binomial regression (Atkins & Gallop, 2007) was applied to examine the effects of excellencism and perfectionism on a severely skewed dependent variable (see Study 3 in Gaudreau et al., 2022). Multilevel modeling would be appropriate for researchers measuring a dependent variable in intensive longitudinal designs across multiple episodes, days, or weeks (e.g., Nezlek, 2012). Alternatively, growth modeling could be used to track the developmental trajectory of a dependent variable over weeks, months, and years (e.g., Grimm & Ram, 2012). Examples of multilevel (e.g., Dunkley, 2018) and growth curve models (e.g., Gaudreau et al., 2019) can be found in the perfectionism literature. All of these approaches offer the flexibility of the variable-centered tradition and can be extended to account for measurement error (i.e., latent variable models) and to investigate the potential effects of mediators and moderators. Regardless of the precise analytical approach, what is currently missing is a theory-driven roadmap to determine how to use the unique/main effects of excellencism and perfectionism to compare the outcomes associated with nonexcellence/nonperfection, excellence, and perfection strivers. Therefore, our goal was to provide this theoretical roadmap in the following nine interpretational scenarios.

### Hypothetical scenarios of excellencism and perfectionism effects

The idea that people who pursue perfection are never satisfied remains a rallying theme in the perfectionism literature (e.g., Egan et al., 2016; Hewitt et al., 2017). Perfectionistic athletes in a recent mixed-method study were described as having an active resistance to contentment (Gotwals & Tamminen, 2022). This is consistent with a tendency to ruminate about mistakes (Flett et al., 2019) and discount the positives (Egan et al., 2016). In this study, we conducted two secondary data analyses based on studies that focused on people’s beliefs about their capacity to savor positive experiences in school (Schellenberg & Gaudreau, 2020) and sport (e.g., Schellenberg, Verner-Filion, et al., 2022). Savoring involves efforts to augment or prolong one’s enjoyment of positive experiences (Bryant & Veroff, 2017). People can savor positive experiences as they are unfolding (savoring the moment), that happened in the past (reminiscence), or that they expect to happen in the future (anticipation). In our hypothetical example, we consider the capacity to savor positive events to be adaptive because savoring has been linked with many beneficial outcomes related to psychological, social, and even physical well-being (for a review, see Bryant, 2021).

**Figure 2.**
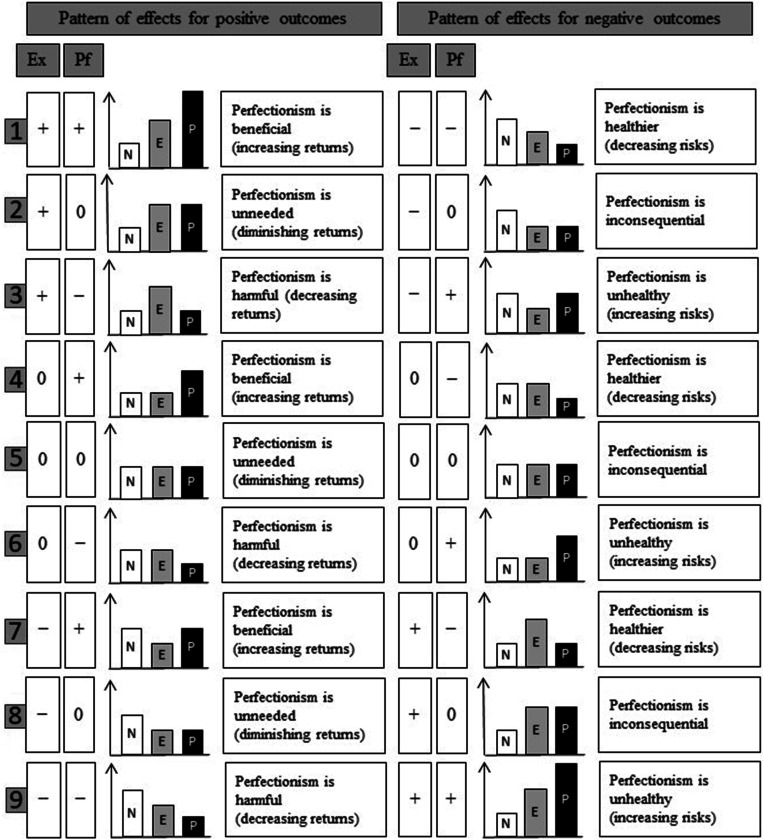
Nine interpretational scenarios for the excellencism and perfectionism effects when predicting positive and negative outcomes. Note. Ex: Excellencism; Pf: Perfectionism; N: Nonexcellence/nonperfection strivers; E: Excellence strivers; P: Perfection strivers; +: Positive Beta; 0: Null Beta; −: Negative Beta. In all cases and interpretations, P is compared to E (rather than N); Horizontal axis in left panel: Predicted values of a positive outcome; Horizontal axis in right panel: Predicted values of a negative outcome.

Imagine a researcher who decides to perform a multiple regression with excellencism and perfectionism as the independent variables to predict savoring (i.e., the dependent variable). Nine interpretational scenarios are possible based on the patterns of unique effects observed for excellencism (i.e., positive, null, or negative) and perfectionism (i.e., positive, null, or negative). These interpretational scenarios are illustrated and explained for positive outcomes (e.g., well-being, performance) and then for negative outcomes (e.g., depression, loneliness) in Figure 2. They offer a roadmap to assess the extent to which empirical data matches the MEP alternative hypotheses that perfectionism is beneficial, unneeded, or harmful (Gaudreau, 2019; Gaudreau et al., 2022).

First, three scenarios of excellencism and perfectionism effects (i.e., scenarios 1, 4, and 7) can be taken to support the hypothesis that *perfectionism is beneficial*. What do we exactly mean by beneficial? Perfectionism goes above and beyond the high standards involved in excellencism and therefore requires additional resources. Perfectionism—but not excellencism—is also associated with the many cognitive (e.g., doubts, concerns), behavioral (e.g., imposing perfectionism on others), and social (e.g., perceived pressure to be perfect) expressions of perfectionism (Gaudreau, 2021; Gaudreau et al., 2022). Therefore, for perfectionism to be beneficial, it needs to be associated with increased returns compared to excellencism. This can be shown when perfection strivers, for example, are happier and more productive than excellence strivers. This can also be shown when they experience lower maladjustment compared to excellence strivers. Scenarios 1, 4, and 7 fulfill this requirement.

Second, three patterns of excellencism and perfectionism effects (i.e., scenarios 2, 5, and 8) are supportive of the hypothesis that *perfectionism is unneeded*. Perfectionism can be interpreted as unneeded when the additional investments beyond the pursuit of excellence are unworthy and cost ineffective. The word unneeded is meant to describe the cases in which the aiming and striving toward perfection are associated with returns or benefits that are comparable (i.e., negligibly different) to those associated with the aiming and striving toward excellence. Perfection striving involves greater investments (e.g., relentless and obsessive strivings) and psychological costs (e.g., concerns, doubts, fears) than striving for excellence. Therefore, we can conclude that excellencism is “good enough” in the three scenarios in which perfectionism yields no or negligible benefits beyond excellencism. In scenarios 5 and 8, we could easily conclude that excellencism is also unneeded because it fails to yield additional returns over and above the pursuit of low standards (e.g., low excellencism/low perfectionism). Excellencism is not a miracle cure and there will be subpopulations, situations, and outcomes for which it yields no or negligible advantage compared to aiming and striving toward lower standards (Gaudreau et al., 2022). Overall, scenarios 2, 5, and 8 indicate that perfection strivers do not have a significantly higher level of psychological adjustment (e.g., happiness, performance) than excellence strivers, and therefore provide support for the hypothesis that perfectionism is unneeded.^2^

Third, three patterns of excellencism and perfectionism effects (i.e., scenarios 3, 6, and 9) can be taken to support the hypothesis that *perfectionism is harmful* (i.e., lower adjustment and/or higher maladjustment). The MEP proposes that the effects of perfectionism should be probed against excellencism rather than non-perfectionism (Gaudreau, 2019). Individuals with lower standards have been shown to manifest lower need for achievement, to pursue easier goals, and to make lower progress in the pursuit of their goals (e.g., Gaudreau et al., 2022). Comparing perfection strivers to those individuals may inflate the impression that perfectionism is associated with desirable outcomes. Being under-invested (i.e., low standards) and over-invested (i.e., perfectionistic standards) may both be detrimental for one’s productivity and psychological adjustment (e.g., scenario 3). When using excellencism as the comparison point, scenarios 3, 6, and 9 indicate that perfection strivers have significantly lower psychological adjustment (e.g., less happiness, poorer performance) than excellence strivers. The same holds true when they have significantly higher maladjustment than excellence strivers. Across these three scenarios, perfectionism is associated with decreasing returns (or increased risks in the case of maladjustment) compared to excellencism, thus fulfilling the requirement to support the hypothesis that perfectionism is harmful.

### This research

The nine scenarios presented in Figure 2 extend the previous theory elaboration of the MEP (Gaudreau, 2019, 2021, Gaudreau et al., 2022, 2023). The current research was designed as a substantial-methodology synergy to illustrate how excellencism and perfectionism effects can be interpreted using the nine different scenarios presented in Figure 2. The results of two studies are presented to demonstrate how the hypotheses of the MEP can be tested across five popular statistical analyses. We also created a Shiny App using R Studio to help researchers to calculate the predicted values and visualize the outcomes associated with nonexcellence/nonperfection strivers, excellence strivers, and perfection strivers. The Shiny App is available at: https://model-of-excellencism-and-perfectionism.shinyapps.io/Shiny_Version2/.

## Study 1

In Study 1, we investigated the associations of excellencism and perfectionism with savoring positive school experiences and school dropout intentions. Our goal was to illustrate how four popular variable-centered statistical analyses can be used to test the hypotheses of the MEP (see Table 1). We take for granted that readers are familiar with these statistical analyses and our goal is to demonstrate how to interpret them in the context of the MEP. We started with a first example using a *multiple regression* with one dependent variable (i.e., total score of savoring). Then, we followed with a second example using *multivariate multiple regression* with three correlated dependent variables (i.e., the three subscales of savoring). We pursued with a third example using *structural equation modeling with latent variables* of excellencism, perfectionism, and savoring. A final analysis was performed on school dropout intention to illustrate the benefits of the *zero-inflated negative binomial regression* (Atkins & Gallop, 2007) to analyze severely skewed negative outcomes like the ones frequently studied in the perfectionism literature (e.g., depression and suicidality).

**Table 1.** Overview of our seven-step approach to test the hypotheses of the MEP.

Step	General description	Adaptations in study 2
# 1	*Data preparation.* Create the scores needed for the analysis. We use standardized scores for the data cleaning and centered scores of the predictors for the main analysis.	After centering the predictors and moderator, create multiplicative scores (excellencism × moderator; perfectionism × moderator) for the moderated regression analysis.
# 2	*Data cleaning.* Outliers detection, filtering participants using honesty/attention checks. Assessment/treatment of missing data.	Exclude the multiplicate terms from the detection of outliers.
# 3	*Run the models.* Report mean and standard deviations of all variables. Report bivariate correlations between all variables. Run the selected multivariable model (e.g., multiple regression).	If excellencism × moderator is significant, calculate simple intercepts and slopes of excellencism at low, medium, and high levels of the moderator. If perfectionism × moderator is significant, calculate simple intercepts and slopes of perfectionism at each level of the moderator.
# 4	*Calculate the predicted values* of nonexcellence/nonperfection, excellence, and perfection strivers using the formulas in the Appendix. This process is streamlined using our Mplus syntax or MEP Shiny App.	Based on simple intercepts and slopes, calculate the predicted values at low, medium, and high levels of the moderator for nonexcellence/nonperfection, excellence, and perfection strivers (see Table 5).
# 5	*Calculate effect size* to quantify the size of standardized difference (Cohen’s *d*) or raw difference (delta method) between the predicted values of nonexcellence/nonperfection, excellence, and perfection strivers. Mplus provides exact *p* value of these differences. 95% CI that do not include zero indicates a significant difference at *p* < .05.	Calculate the effect size at low (Panel A of Figures 5 and 6), medium (Panel B of Figures 5 and 6), and high levels (Panel C of Figures 5 and 6) of the moderator.
# 6	*Create a figure* to display the predicted values of nonexcellence/nonperfection, excellence, and perfection strivers for each dependent variable with the MEP Shiny App.	Using the MEP Shiny App, three different figures are created at low, medium, and high levels of the moderator (see Figures 5 and 6).
# 7	*Interpret* the results based on the 9 interpretational scenarios presented in Figure 2.	Interpret the results to consider how the differences between nonexcellence/nonperfection, excellence, and perfection strivers vary depending on the levels of the moderator.

Note. The MEP Shiny App is available at: https://model-of-excellencism-and-perfectionism.shinyapps.io/Shiny_Version2/.

### Method

#### Participants

This sample is based on the 282 participants who reported their savoring experience at school as part of a larger project on passion and savoring (Schellenberg & Gaudreau, 2020) and was also used in the validation process of the Scale of Perfectionism and Excellencism (Gaudreau et al., 2022; see Study 1 sample 2). Based on a priori power analysis in G*Power, this sample size was sufficiently powered to reject null hypothesis (*p* < .05, power 80%) of typical effects observed in personality psychology (Bosco et al., 2015; Gignac & Szodorai, 2016) and expected to be found using the MEP (e.g., β = .16; see Study 2 from Gaudreau et al., 2022). Seven and four participants were respectively excluded because they missed a quality check question included in the questionnaire and were found to be multivariate outliers (χ^2^ = 22.46, *df* = 6, *p* < .001).^3^ In the final sample (*N* = 271), a majority of participants were female (72.3%) and 47.2% described themselves as White/European. They were between 16 and 47 years old (*M* = 19.70 years, *SD* = 3.46 years) with 96% of the sample ranging between 17 and 25 years of age. School was considered important for a majority of them (*M* = 5.08 out of 7; 84% of the participants reported a score of 4 and above). The study was approved by the research ethics committee at the University of Ottawa (Protocol #H12-16-19) and all participants consented to participate.

#### Design and measures

We conducted an online survey with a cross-sectional design. Participants were recruited in an undergraduate students’ pool of participants and they received 1% toward their introduction to psychology class.

Excellencism and perfectionism were measured with the Scale of Perfectionism and Excellencism (SCOPE; Gaudreau et al., 2022). The SCOPE contains 22 items and asks participants to evaluate the extent to which each item corresponds to goals they have in their lives in general using a 1 (*not at all*) to 7 (*totally*) rating scaling. Eleven items are measuring excellencism (“My general goal in life is to perform very well”) and perfectionism (“My general goal in life is to be a flawless person”), respectively. Results of exploratory and confirmatory factor analyses provided support for the bidimensional structure and composite reliability of the scores from the SCOPE (Gaudreau et al., 2022).

Savoring was measured with the Savoring Beliefs Inventory (SBI), which contains 24 items divided in three subscales: anticipatory savoring (“Before a good thing happens, I look forward to it in ways that give me pleasure in the present”), momentary savoring (“I feel fully able to appreciate good things that happen to me”), and reminiscence savoring (“I enjoy looking back on happy times from my past”). In this study, participants were asked to think about positive events they experienced in their academic lives and to rate the SBI items on a scale from 1 (*not at all true*) to 7 (*totally true*). Results from bifactor confirmatory factor analyses showed that the scores of the SBI can be analyzed using either the individual subscales or a total score (e.g., Bryant, 2003; Bryant, 2021; Kawakubo, 2019).

Finally, this study included three items related to dropout intentions (“I considered dropping out of university”; “I had some intentions to drop out of university”; “I thought that I would be happier by quitting the university”). Each item was rated on a scale from 0 (*never*) to 6 (*always*). We used the second item in our analyses because it specifically referred to the concept of intention. More importantly, a majority of participants (57%) answered zero on this item, thus making it a very good candidate for our demonstration of the zero-inflated negative binomial regression.

#### Plan of analyses

Analyses were performed in Mplus 8.7 (Muthén & Muthén, 2022). Table S1 of our supplementary file presents an overview to help navigate across the syntax codes, outputs, figures of predicted values, and the Shiny App across the five analyses performed in our two studies. All annotated syntax codes and outputs are publicly available: https://osf.io/8fb49/.

Our analyses were not preregistered but followed the same plan of analyses used in previous MEP research (e.g., Gaudreau et al., 2022). Table 1 summarizes a seven-step approach to test the hypotheses of the MEP. First, we created the scores needed for the analysis. We created composite scores of excellencism, perfectionism, and savoring for the first two analyses (i.e., multiple regression and multivariate multiple regression) and the fourth analysis (i.e., zero-inflated binomial regression). We created parcels of excellencism and perfectionism for the third analyses (i.e., structural equation model). We mean centered the scores of the predictors (composite scores, parcels) to facilitate the calculation of predicted values of the dependent variables. To center the scores, we subtracted the score of each person by the mean of the sample. Second, we started with data cleaning for which we used the Mahalanobis distance (*p* < .001) because the inclusion of multivariate outliers could potentially distort the line of best fit and bias the parameter estimates of our models. Sensitivity analyses (rerunning the models with and without the outliers) are reported in Table S2 and Table S3 of the supplementary file. Third, we estimated the correlations between all variables of our analyses and we ran the selected multivariable analysis. In all analyses, we relied on the full maximum likelihood robust estimator to handle the missing data and to correct the standard error of the parameter estimates for non-normality of the data.

Fourth, using the formulates in the appendix (Cohen et al., 2003), we calculated the predicted value of the dependent variable using the intercept, the unstandardized value of the beta coefficients, and the following values for the predictors: (a) nonexcellence/nonperfection strivers (−1*SD* of excellencism, −1*SD* of perfectionism), (b) excellence strivers (+1*SD* of excellencism, −1*SD* of perfectionism), and (c) perfection strivers (+1*SD* of excellencism, +1*SD* of perfectionism). Predicted values of nonexcellence/nonperfection, perfection, and excellence strivers are calculated using a pick-a-point approach (i.e., −1*SD* and +1*SD*). This approach is largely consistent with the operational definitions advanced in the MEP. Our approach places excellencism and perfectionism as two dimensions with their own continuous distributions; we do not categorize people into subgroups (e.g., Streiner, 2002). Values of −1*SD* and +1*SD* from the mean could be misinterpreted as being not sufficiently extreme. However, approximately 68% of people in a normal distribution are within −1*SD* and +1*SD* from the mean. With a score at −1*SD* of the mean of perfectionism (*M* = 2.55), a person like Jean would be ranked at the 16^th^ percentile of a distribution. With a score at +1*SD* of the mean of perfectionism (*M* = 5.79), a person like Jordan would be ranked at the 84^th^ percentile of a distribution. In our sample, these values corresponded to the 19^th^ and 78^th^ percentile because real-life empirical data generally exhibit slight deviations from a normal distribution. This gives an estimate of how many people are situated beyond the specific points. These points are sufficiently “extreme” to properly define nonexcellence/nonperfection, perfection, and excellence strivers.

Using this approach, the predicted values are calculated using points that are sample specific. This remains adequate in most empirical situations in which the estimates of a sample (*M* and *SD*) are sufficiently comparable with the parameters (i.e., the norms) of the population. This requires nuanced interpretations in situations in which participants are selected on the basis of their elevated perfectionism (e.g., clinical interventions) or when the mean and/or standard deviation of a sample sharply deviate from the norms. It is important to understand that the significance testing (i.e., determining if excellence strivers have higher savoring than perfection strivers) relies on the significance of the beta estimates rather than a median split that would artificially categorize people into subgroups. Therefore, the significance testing and the effect size of the difference between excellence and perfection strivers are derivatives of the beta estimates; they are equivalent across the distribution of the scores and are not influenced by the specific points taken to calculate the predicted values.

Fifth, we calculated a standardized effect size (approximation of a Cohen’s *d*) by dividing the difference between two predicted values by the *SD* of the dependent variable (e.g., Gaudreau, 2012; Hill et al., 2020). This helped to gauge the size of the differences between pairs of predicted values. For the zero-inflated negative binomial regression, the delta method was used to estimate the raw difference (and their statistical significance) between two predicted values (Raykov & Marcoulides, 2004). Both the standardized (Cohen’s *d*) and raw differences (delta) are accompanied with an exact *p* value in Mplus. We also reported the 95% CI of both the standardized and raw effect size to determine if the predicted values significantly differed (*p* < .05) across nonexcellence/nonperfection strivers, excellence strivers, and perfection strivers. All of these analyses are programmed in our Mplus syntax codes and require no manual calculation. For researchers who are not using Mplus, the predicted values and effect size can also be calculated using our online Shiny App.^4^ The 95% CI of Cohen’s *d* can be approximated using online calculators (e.g., Lenhard & Lenhard, 2016).

Sixth, we created figures of the predicted values to facilitate interpretation of the effects. Our figures were automatically generated using the MEP Shiny App. We did not include error bars because overlapping 95% intervals of the predicted values are often mistakenly used to conclude that a difference is statistically non-significant (Cumming & Finch, 2005; Goldstein & Healy, 1995; Knol et al., 2011; Schenker & Gentleman, 2001). Our Shiny App contains the option to display error bars based on adjusted confidence intervals (Cousineau, 2017) calculated using 83.4% CI (Knol et al., 2011). As explained in the supplementary file, inferential statistics and adjusted error bars may result in different interpretations. As summarized by Schenker and Gentleman (2001), “although the method of examining overlap is simple and especially convenient when lists or graphs of confidence intervals have been presented, we conclude that it should not be used for formal significance testing” (p. 182). Therefore, our hypothesis testing is based on inferential statistics (see step 5) rather than visual inspection of the error bars.

Seventh, we compared the results with our nine interpretational guidelines/scenarios (see Figure 2) to determine if perfectionism is beneficial, unneeded, or harmful. Our MEP Shiny App and Mplus syntaxes are not obligatory to test the MEP, but they were designed to streamline this process, enhance clarity, and minimize the chances of misinterpretation.

To conclude, it is important to explain why we relied on the variable-centered approach compared to group-based statistics. Cluster analyses and latent profile modeling work under the assumption that a population is heterogeneous and dividable in unobserved subpopulations (Muthén & Muthén, 2000; Nagin, 1999). Regrouping people in latent categories is complex and the number, definition, and theoretical interpretability of the groups often vary across studies (e.g., Hill & Madigan, 2017). The exploratory and data-driven nature of the group-based approach make it difficult to build a comparable corpus of knowledge across studies. Furthermore, individual differences in perfectionism have been found to be continuous/dimensional rather than taxonic/categorical (Broman-Fulks et al., 2008). As a result, the MEP was developed within the confines of the variable-centered approach. This is consistent with other theories in psychology (Gaudreau, 2023; Schellenberg et al., 2019; Shaver & Mikulincer, 2008) which use a linear combination of the predictors to estimate and compare the predicted outcome of people with different sets of scores on each of the predictors. For all of these reasons, we recommend that researchers test the hypotheses of the MEP using variable-centered statistics.

### Results

#### Preliminary analyses

Studies using the MEP should expect mean scores of excellencism (*M* = 5.87, *SD* = .90) to be higher than perfectionism (*M* = 4.17, *SD* = 1.62) and moderately correlated (*r* = .49). These descriptive statistics are similar to previous studies on the SCOPE who reported correlations that varied between .35 and .51 (Gaudreau et al., 2021, 2022; Goulet-Pelletier et al., 2022). Means, standard deviations, internal consistency, and bivariate correlations are reported in Table 2.

**Table 2.** Study 1 descriptive statistics and bivariate correlations.

Variables	*M*	*SD*	1	2	3	4	5	6	7
1. Excellencism	5.871	.901	.943						
2. Perfectionism	4.172	1.623	.493**	.972					
3. Savoring	5.204	.810	.287**	−.051	.881				
4. Anticipatory savoring	5.196	.941	.283**	−.067	.891**	.740			
5. Momentary savoring	5.037	.898	.208**	−.009	.857**	.617**	.707		
6. Reminiscence savoring	5.379	.907	.268**	−.057	.906**	.740**	.667**	.724	
7. Dropout intention	1.053	1.471	−.189**	.034	−.317**	−.234**	−.288**	−.322**	n/a

Note. Omega reported on the diagonal. n/a because this is a single item.

**p < .01. *p < .05.

#### Example 1: Multiple regression with one dependent variable

**Figure 3.**
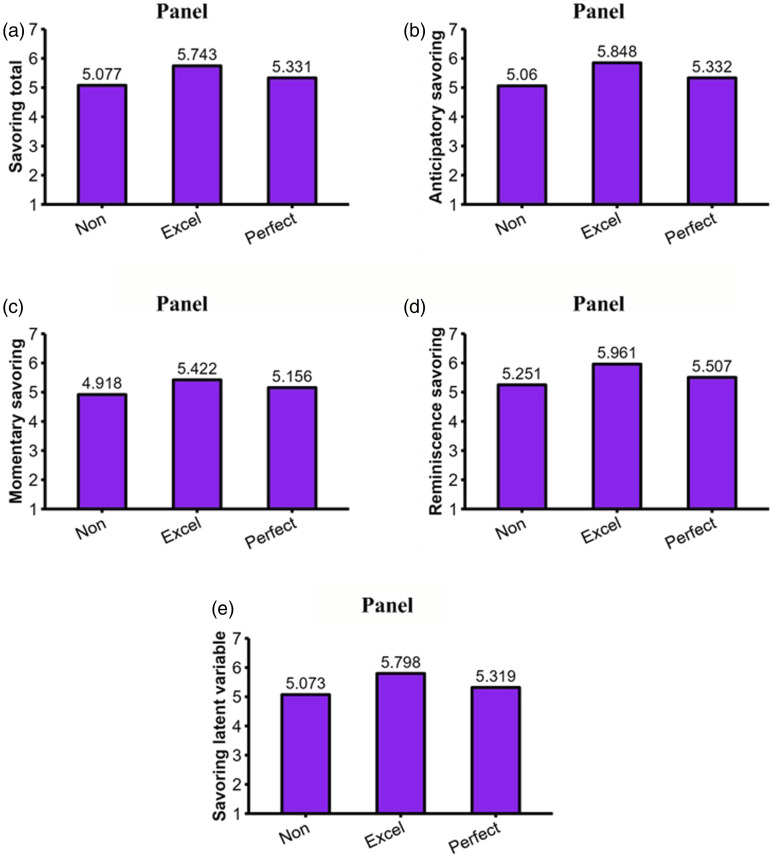
Results of multiple regressions (Panel A), multivariate multiple regression (Panel B–D), and structural equation modeling (Panel E). Note. Non: Nonexcellence/nonperfection strivers; Excel: Excellence strivers; Perfect: Perfection strivers.

In the first example, we used a linear multiple regression with excellencism and perfectionism as independent variables and the total score of savoring as a dependent variable. Results showed that excellencism and perfectionism were positively and negatively associated with savoring, respectively. The predictors explained 13.1% of the variance in savoring (see Table 3). The predicted values displayed in Figure 3 (panel A) revealed that excellence strivers savor their positive academic experience to a larger extent compared to both nonexcellence/nonperfection strivers and perfection strivers, whereas perfection strivers savored to a larger extent than nonexcellence/nonperfection strivers. Differences were consequential with effect size (*d*) all above |.337|. Interpretational scenario #3 of the MEP (see Figure 2) was the one that was the most closely aligned with this pattern of excellencism and perfectionism effects.

**Table 3.** Study 1 associations of excellencism and perfectionism with savoring using three different analyses.

Analyses/Dependent variables	Intercept	Excellencism			Perfectionism			*R* ^2^
		B	*SE*	*β*	B	*SE*	*β*	
Multiple regression								
Savoring – composite score	5.204	.370	.052	.412**	−.127	.033	−.254**	.131
Multivariate multiple regression								
Anticipatory savoring	5.196	.437	.062	.418**	−.159	.038	−.274**	.137
Momentary savoring	5.037	.280	.060	.281**	−.082	.039	−.148*	.060
Reminiscence savoring	5.379	.394	.058	.391**	−.140	.038	−.250**	.119
Structural equation modeling								
Savoring – latent variable	5.196	.452	.071	.461**	−.164	.041	−.304**	.167

Cohen’s d effect size with 95% CI^a^			
	Excel versus Non	Perfect versus Excel	Perfect versus Non
Savoring – composite score	.822** [.60, 1.05]	−.508** [−.77, −.25]	.316* [.06, .54]
Anticipatory savoring	.837** [.60, 1.07]	−.548** [−.81, −.29]	.289* [.03, .55]
Momentary savoring	.562** [.32, .80]	−.295* [−.57, −.02]	.267 [−.01, .54]
Reminiscence savoring	.782** [.56, 1.01]	−.500** [−.77, −.23]	.282* [.04, .52]
Savoring – latent variable	.923** [.62, 1.22]	−.608** [−.91, −.30]	.315* [.02, .61]

Note. *N* = 271.

Excel: Excellence strivers; Perfect: Perfection strivers; Non: Not pursuing excellence nor perfection.

**p < .01. *p < .05.

^a^Effect size is approximative and they are calculated by subtracting two predicted values before dividing it by the *SD* of the dependent variable (values reported here are taken from Mplus).

#### Example 2: Multivariate multiple regression with three dependent variables

The second example is less frequently used in psychological science. Multivariate multiple regression allows the simultaneous modeling of several dependent variables. Correlated dependent variables may or may not share exactly the same associations with excellencism and perfectionism. Savoring can be divided in three temporal perspectives. In our example, anticipatory savoring, momentary savoring, and reminiscence savoring were modeled as correlated dependent variables each predicted by excellencism and perfectionism.

Results showed that excellencism and perfectionism were respectively positively and negatively associated with anticipatory savoring, momentary savoring, and reminiscence savoring (see Table 3). The predictors explained 13.7%, 6%, and 11.9% of the variance in each of the three temporal perspectives on savoring. The predicted values displayed in Figure 3 (panels B to D) revealed that excellence strivers engaged in anticipatory savoring, momentary savoring, and reminiscence savoring to a larger extent compared to both nonexcellence/nonperfection strivers and perfection strivers, whereas perfection strivers engaged in anticipatory savoring and reminiscence savoring to a larger extent than nonexcellence/nonperfection strivers. Differences were consequential with effect size (*d*) all above |.301|. Interpretational scenario #3 of the MEP (see Figure 2) was the one that was the most closely aligned with this pattern of excellencism and perfectionism effects.

We had no specific hypothesis about potential differences in the associations of excellencism and perfectionism with the three dependent variables of the model. However, we used this example to illustrate how such hypotheses could be directly tested using the delta method (Raykov & Marcoulides, 2004). We compared the beta of perfectionism with momentary savoring to the betas of perfectionism with anticipatory savoring and reminiscence savoring. The perfectionism effect was less negative for momentary savoring compared to anticipatory savoring (Δ_*betas*_ = .077, *SE* = .035, *p* = .027), but the difference between momentary savoring and reminiscence savoring was not statistically significant (Δ_*betas*_ = .058, *SE* = .034, *p* = .084). The capacity to directly compare the effect of a predictor across different dependent variables is a definitive strength of the multivariate multiple regression model.

#### Example 3: Structural equation modeling with latent variables

In our third example, we presented a structural equation model (SEM) in which excellencism, perfectionism, and savoring were conceived using multiple indicators to create latent variables. Latent variables are useful to estimate the effects between “true score” or associations between variables that are not attenuated by measurement error (Fan, 2003). SEM can be performed using items of questionnaires, parcels (i.e., average of several items), or composite scores (i.e., subscales of a questionnaire). Different approaches have their strengths and weaknesses. In the case of the SCOPE, using the 22 items as indicators would be challenging and unrealistic (e.g., too many parameters to be estimated), especially when modeling the associations of excellencism and perfectionism with other external criteria. In this study, we created four parcels for excellencism and four parcels for perfectionism (Gaudreau et al., 2022, see Study 5). In the case of savoring, we relied on the three subscales of the savoring beliefs inventory to create a latent variable of savoring. The first indicator was fixed to 1 to identify each of the latent variables.

The model can be divided in two parts: Measurement and structural. At the measurement level, the results provided good evidence for the factorial structure of the model: MLR χ^2^ = 121.725, *df* = 41, *p* < .001, CFI = .965, TLI = .953, SRMR = .040, RMSEA = .085, RMSEA 90% CI = [.068, .103]. The standardized factor loadings were high for the parcels of excellencism (average λ = .877, range from .841 to .904), parcels of perfectionism (average λ = .935, range from .908 to .965), and subscales of savoring (average λ = .822, range from .741 to .889). The composite reliability of the excellencism (ω = .930), perfectionism (ω = .965), and savoring (ω = .863) latent variables provided evidence for the internal consistency of the latent scores (see online calculator: https://www.thestatisticalmind.com/composite-reliability/). The variance of the three latent variables (needed to calculate the predicted values in the structural model) were excellencism (σ^2^ = .643, *SD* = .802), perfectionism (σ^2^ = 2.126, *SD* = 1.458), and savoring (σ^2^ = .618, *SD* = .786).

At the structural level, the intercept of the first indicator of the dependent variable was fixed to 0. That way, the intercept of the latent dependent variable can be used to calculate and interpret the predicted values on the 1 to 7 scale that we used to measure savoring.^5^ Results showed that excellencism and perfectionism were positively and negatively associated with savoring, respectively (see Table 3). The predictors explained 16.7% of the variance in savoring. The predicted values displayed in Figure 3 (panel E) revealed that excellence strivers savor their positive academic experience to a larger extent compared to both nonexcellence/nonperfection strivers and perfection strivers, whereas perfection strivers savored to a larger extent than nonexcellence/nonperfection strivers. Interpretational scenario #3 of the MEP (see Figure 2) was the one that was the most consistent with this pattern of excellencism and perfectionism effects.

#### Example #4: Zero-inflated negative binomial regression model

**Figure 4.**
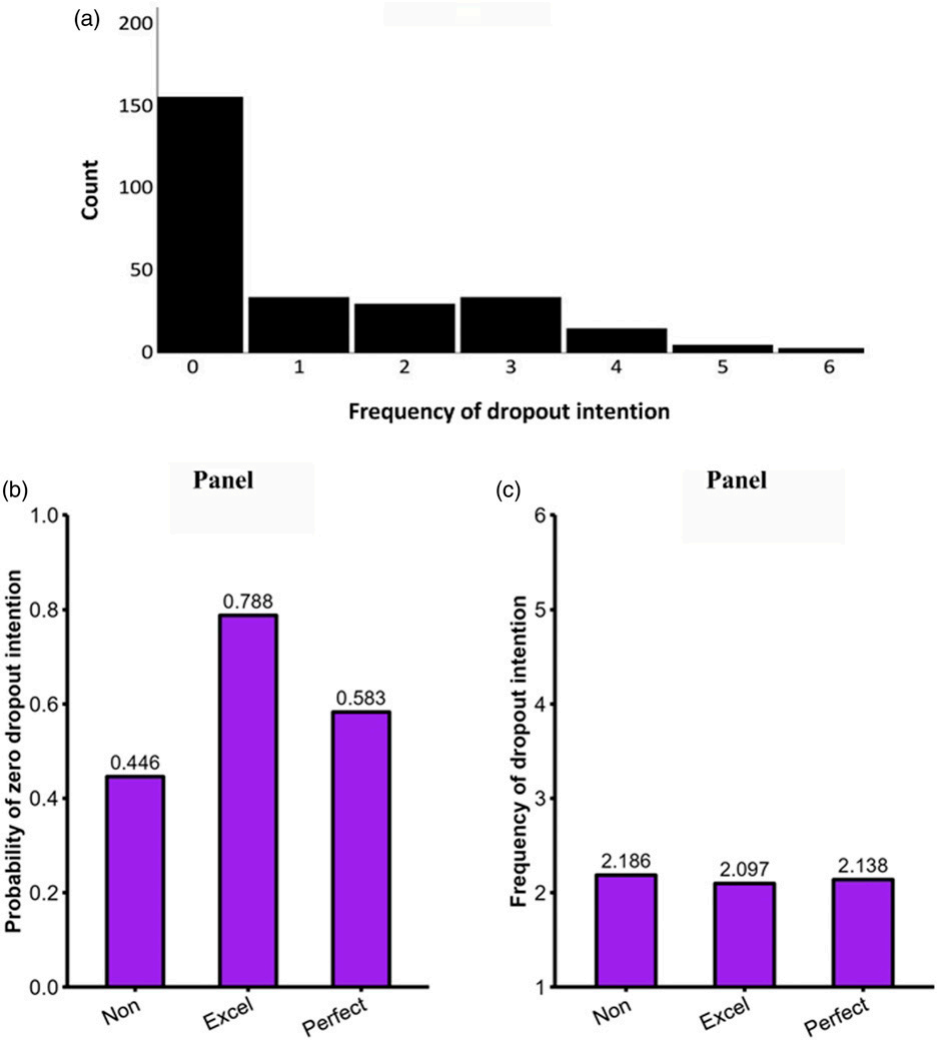
Distribution of dropout intention (Panel A) and results of the zero-inflated negative binomial regression (Panel B–C). Note. Non: Nonexcellence/nonperfection strivers; Excel: Excellence strivers; Perfect: Perfection strivers.

For our last example, we turned our attention to the dropout intention variable. The distribution of the dropout intention scores (*M* = 1.053, *SD* = 1.471, mode = 0) is shown in panel A of Figure 4. Most students (153 out of 269)^6^ never had the intention to dropout from school. This zero-mode distribution with overly dispersed scores (mean is lower than the variance of 2.163) violates the assumptions of regular multiple regressions. A *zero-inflated negative binomial regression model* (e.g., Atkins & Gallop, 2007) is a good approach to properly estimate the effects of excellencism and perfectionism in these situations. This model combines the features and advantages of logistic and regular multiple regression. Dropout intention is divided in (a) a binary component (0 = “*never”*; 1 = some level of dropout intention) and (b) a continuous component (i.e., dropout intention from 1 “*very rarely”* to 6 “*always”*). Therefore, the model combines (a) a logistic regression equation to predict the zero-class or the probability of never thinking about dropping out and (b) a count regression equation to predict the frequency of dropout intentions. Predicted values for both components of the model are automatically calculated in our Mplus syntax and Shiny App using the formulas presented in the Appendix.

*The probability of belonging to the zero-class* (i.e., never thinking about dropping out; Intercept = .059, *S.E.* = .177, *p* = .737) was significantly predicted by excellencism (B = .850, *S.E.* = .217, *p* < .001, *β* = .395) and perfectionism (B = −.300, *S.E.* = .101, *p* = .003, *β* = −.252). As shown in Figure 4 (panel B), the probability of never thinking about dropout was higher for excellence strivers compared to nonexcellence/nonperfection (raw difference = .342, 95% CI = [.189, .495]) and lower for excellence strivers compared to perfection strivers (raw difference = −.205, 95% CI = [−.328, −.081]). The difference between perfection strivers and nonexcellence/nonperfection strivers was not significant (raw difference = .137, 95% CI = [−.051, −.325]). This component of the model can be graphed using the binary logistic regression section of the MEP Shiny App.

*The frequency of dropout intention* (Intercept = .771, *S.E.* = .095, *p* < .001) was not significantly predicted by excellencism (B = −.023, *S.E.* = .091, *p* = .803, *β* = −.020) and perfectionism (B = .006, *S.E.* = .057, *p* = .918, *β* = .009). Among those who sometimes think about dropping out (see Figure 4, panel C), there was no significant difference in the frequency of dropout intentions. Excellence strivers did not differ from nonexcellence/nonperfection (raw difference = −.087, 95% CI = [−.758, .584]) and perfection strivers (raw difference = .040, 95% CI = [−.720, .800]), which also did not differ from nonexcellence/nonperfection strivers (raw difference = −.047, 95% CI = [−.760, .667]). This component of the model can be graphed using the zero-inflated binomial section of the MEP Shiny App.

### Brief discussion

Both savoring and having zero intention to dropout from school were positively and negatively associated with excellencism and perfectionism, respectively. Overall, the third interpretational scenario of the MEP (see Figure 2) was the one that most closely matched the results with savoring and the probability of having zero intention to dropout from school.

## Study 2

Our aim was to extend the analyses of Study 1 in four important ways. First, Study 1 relied on a cross-sectional design that may have overestimated the effects of excellencism and perfectionism as well as the differences between nonexcellence/nonperfection, excellence, and perfection strivers. Second, our results were limited to university students and we therefore turned our attention to a sample of college-aged athletes at the start and in the middle of their competitive season. Third, not only did we try to replicate our previous findings with savoring, but we also investigated athletes’ enjoyment and fun playing their sport (i.e., sport enjoyment). Finally, moderators are often proposed but unfrequently tested in the perfectionism literature. In Study 2, we revisited the moderating role of goal attainment.

Goals are self-regulatory standards used to evaluate task mastery, progress, and success relative to others (e.g., Bandura, 1997; Carver & Scheier, 1998; Elliot et al., 2011). A well-established finding across self-regulation theories is the idea that people feel bad after failing to attain a goal. A rallying point in the perfectionism literature is the idea that the emotional costs of failure are higher for people who pursue perfection (e.g., Crocker et al., 2014; Curran & Hill, 2018; Egan et al., 2016; Hewitt et al., 2017). Perfection strivers have elevated fear of failure (Conroy et al., 2007). They are concerned about mistakes and they entertain doubts about their actions (Frost et al., 1990). They are critical of themselves (Blatt, 1995) and their self-worth is contingent on their capacity to attain their unrealistic and extremely high standards (Egan et al., 2016; Hewitt et al., 2017). All of this creates a dialectic that can transform goal achievement (an otherwise desirable outcome) into an obligatory condition for self-worth and happiness. Without goal attainment, perfection strivers are unlikely to feel satisfied about themselves. As such, it can be assumed that perfectionistic athletes savor and enjoy their sport experience only to the extent that they reach high levels of goal attainment. This is consistent with the position that achievement-related difficulties are more stressful for people who pursue perfection (e.g., Flett et al., 1995). Goal-related difficulties (Flett & Hewitt, 2014, 2019), failures (Curran & Hill, 2018), and chronic distress or adverse environments (Gaudreau et al., 2018) can aggravate the negative outcomes of perfectionism. For example, perfection strivers (compared to non-perfectionism) have been found to experience more perceived control and positive affect (Crocker et al., 2014) and less tensed arousal after a competition (Waleriańczyk et al., 2022), but these effects were limited to athletes with high levels of goal attainment in their sport. When performers attain their goals, perfectionism is likely to appear just as good as excellencism. When they fail to attain their goals, perfection strivers are likely to experience harmful psychological outcomes compared to excellence strivers.

In this study, we assumed that the savoring and sport enjoyment of the perfection strivers are potentially contingent on achievement. Under low and medium levels of goal attainment, we can expect perfection strivers to experience significantly lower savoring and sport enjoyment compared to excellence strivers. Only if they reach a high level of goal attainment should the perfection strivers experience the savoring and sport enjoyment typically experienced by excellence strivers. We tested the associations of excellencism and perfectionism measured at the start of the season (Time 1) with savoring and enjoyment measured in the middle of the season (Time 2).

### Method

#### Participants

This sample is based on the 298 participants who participated in the Student-Athlete Well-Being and Achievement Project (Schellenberg et al., 2023; Schellenberg et al., in press; Schellenberg, Semenchuk, et al., 2022; Schellenberg, Verner-Filion, et al., 2022). A priori power analysis was conducted in G*Power and this sample size was sufficiently powered to reject null hypothesis (*p* < .05, power 80%) of typical effects observed in personality psychology (e.g., β = .16) and similar to the ones observed in Study 1. Two participants were excluded because they were found to be multivariate outliers (χ^2^ = 24.32, *df* = 7, *p* < .001). In the final sample (*N* = 296), a majority of participants were male (56%) and 62.1% described themselves as White/European. They were between 17 and 35 years old (*M* = 20.10, *SD* = 2.43) with 97% of the sample ranging between 17 and 25 years of age. They participated in four different varsity sports (i.e., hockey, basketball, volleyball, and track and field). On average, they participated in their sport 16 hours per week, which denotes a high level of involvement in their sport. Sport was considered important for a majority of them (*M* = 6.20 out of 7; 94% of the participants reported a score of 5 and above). The study was approved by the education/nursing research ethics committee at the University of Manitoba (Protocol #E2019:046) and all participants consented to participate. Another survey completed at the end of the season is not analyzed in this study. Participants received a 5$ CAD compensation at Time 1 and a gift certificate at the end of the study.

#### Design and measures

Participants completed the SCOPE at the start of the season (September-October; Time 1) to measure excellencism and perfectionism. In the middle of the season (December; Time 2), they completed the SBI to measure savoring. They were asked to think of how they generally feel about positive events that occur while they are playing in their sport. A third survey near the end of the season (February) is not included in this study but is fully described in our OSF link to material and data. Rating scales from the SCOPE and SBI were identical to Study 1.

Participants also reported on their sport enjoyment (Scanlan et al., 1993). For each of the four items (e.g., “Do you enjoy playing your sport?”) they were asked how they have been feeling playing their sport over the past four weeks using a five-point scale from 1 (*not at all*) to 5 (*very much*). Finally, they evaluated their level of goal attainment using the Attainment of Sport Achievement Goal Scale (Amiot et al., 2004). Each of the 12 items was rated on a 1 (*not at all*) to 7 (*totally*) scale to capture athletes’ subjective evaluation of their mastery-based (i.e., executed my movements correctly”), normative-based (i.e., “outperformed other athletes”), and improvement-based (i.e., “I did better than my usual performances”) level of goal attainment in their sport over the last four weeks. The three subscales were correlated (mastery-normative, *r* = .529, mastery-improvement, *r* = .643; improvement-normative, *r* = .654) and meant to produce a global evaluation of goal attainment or subjective sport achievement (e.g., Martinent et al., 2013).

#### Plan of analyses

Two moderated multiple regressions were performed using Time 2 savoring and Time 2 sport enjoyment as dependent variables (see example 5 in Table S1). Excellencism, perfectionism, and goal attainment were centered and entered as independent variables. Multiplicative terms were created to test two interactive effects: excellencism × goal attainment and perfectionism × goal attainment. Because perfectionism and excellencism share a partially conjunctive association (i.e., perfectionistic pursue excellence but they also pursue perfection; see Gaudreau, 2019, Figure 1), tests of the MEP should not involve an interaction between excellencism and perfectionism nor a three-way interaction. A significant interaction term was followed-up by estimating the association of a predictor (i.e., perfectionism) at low (−1*SD*), medium (average of the sample), and high (+1*SD*) values of goal attainment. These simple intercepts and slopes were used to calculate the predicted values of each outcome and their standardized differences (Cohen’s *d*) at low, medium, and high levels of goal attainment for (a) nonexcellence/nonperfection strivers (−1*SD* of excellencism, −1*SD* of perfectionism), (b) excellence strivers (+1*SD* of excellencism, −1*SD* of perfectionism), and (c) perfection strivers (+1*SD* of excellencism, +1*SD* of perfectionism). All of these analyses are programmed in our Mplus syntax codes and require no manual calculation. For users who are not using Mplus, the predicted values and effect size can also be calculated using our online MEP Shiny App. Figures of the predicted values are automatically generated using the MEP Shiny App.

### Results

#### Preliminary analyses

A sample of 126 (out of 296) completed the Time 2 survey. Those of completed Time 2 did not differ significantly from those who did not on excellencism, *t* (294) = .328, *p* = .743, and perfectionism, *t* (294) = .970, *p* = .333. All participants were included in the analyses and the missing data were handled with full maximum likelihood estimation.

Mean score of excellencism in this sample was higher than Study 1 and a previous study with sport participants (Gaudreau et al., 2021), but still comparable to some other studies on the MEP (see Study 5 in Gaudreau et al., 2022). Excellencism (*M* = 6.20, *SD* = .78) was higher than perfectionism (*M* = 4.24, *SD* = 1.68) and moderately correlated (*r* = .46). Mean, standard deviation, and internal consistency of each variable are presented in Table 4 along with their bivariate correlations.

**Table 4.** Study 2 descriptive statistics and bivariate correlations.

Variables	M	SD	1	2	3	4	5
1. Time 1 excellencism	6.204	.776	.942				
2. Time 1 perfectionism	4.237	1.675	.461**	.973			
3. Time 2 savoring	4.919	.707	.139	−.130*	.864		
4. Time 2 enjoyment	4.393	.638	.411**	.069	.334**	.935	
5. Time 2 goal attainment	3.942	1.049	.168**	.237**	.081	.280**	.923

Note. *N* = 296. Omega reported on the diagonal.

**p < .01. *p < .05.

#### Moderated multiple regression

Results are presented in Table 5. Goal attainment was significantly associated with higher sport enjoyment, but not with savoring. Even while accounting for goal attainment and its interactive effects, excellencism and perfectionism were positively and negatively associated with both savoring and sport enjoyment, respectively. Overall, these findings replicated and extended those of Study 1.

**Table 5.** Study 2 associations of excellencism and perfectionism with savoring and enjoyment moderated by goal attainment.

	Time 2 savoring			Time 2 enjoyment		
	B	*SE*	*β*	B	*SE*	*β*
Intercept	4.871	---	---	4.373	---	---
Excellencism (E)	.218*	.091	.237	.373**	.068	.436
Perfectionism (P)	−.118**	.040	−.276	−.076*	.030	−.190
Goal attainment (GA)	.070	.054	.102	.169**	.052	.265
E × GA	.050	.083	.051	−.130	.082	−.142
P × GA	.062*	.028	.166	.054*	.027	.157

Simple slopes				
@ Low goal attainment				
Intercept	4.798	---	4.195	---
Excellencism	.218*	.091	.373**	.068
Perfectionism	−.183**	.042	−.133**	.042
@ High goal attainment				
Intercept	4.944	---	4.551	---
Excellencism	.218*	.091	.373**	.068
Perfectionism	−.054	.057	−.019	.041

Note. *N* = 296. Standardized estimates are not available for simple slopes.

**p < .01. *p < .05.

The excellencism × goal attainment interaction did not reach statistical significance, thus indicating that the associations between excellencism and both savoring and sport enjoyment did not significantly differ across levels of goal attainment. These associations were held constant across the three levels of goal attainment in our calculation of the predicted values. However, the perfectionism × goal attainment interaction term significantly predicted both savoring and sport enjoyment. Simple slopes indicated that the negative associations between perfectionism and both savoring and sport enjoyment (i.e., significant at low and medium levels of goal attainment) became non-significant at high level of goal attainment (see Table 5).

**Figure 5.**
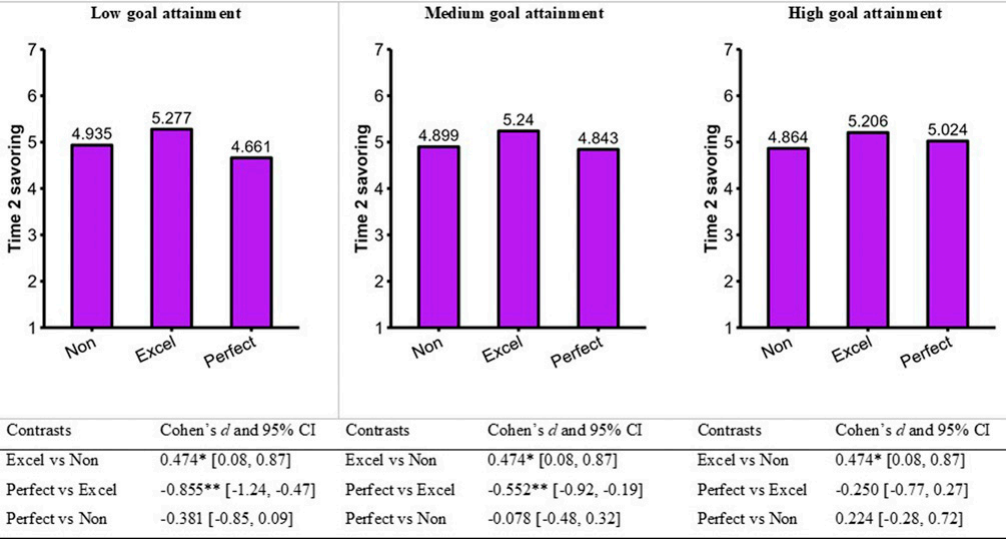
Moderating role of goal attainment for Time 2 savoring. Note. **p < .01. *p < .05. Cohen’s *d* and their 95% CI are exact values taken from Mplus.

**Figure 6.**
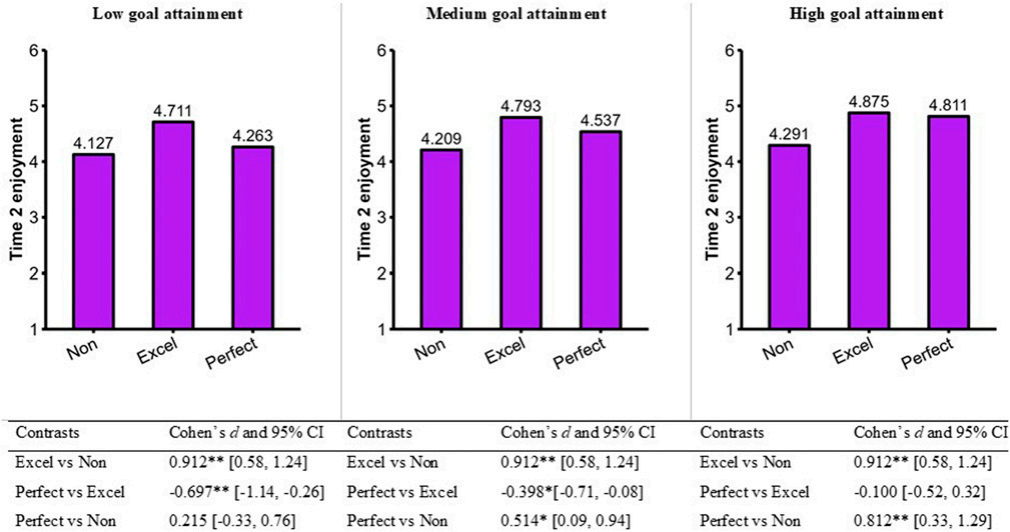
Moderating role of goal attainment for Time 2 enjoyment. Note. **p < .01. *p < .05. Cohen’s *d* and their 95% CI are exact values taken from Mplus.

Based on these estimates, we calculated the predicted values of savoring (see Figure 5) and sport enjoyment (see Figure 6). Excellence strivers savored their positive sport experience and enjoyed their sport to a larger extent compared to nonexcellence/nonperfection strivers. These effects were constant across all levels of goal attainment. Perfection strivers savored their positive sport experience and enjoyed their sport to a lesser extent compared to excellence strivers at lower and medium levels of goal attainment. However, the perfection strivers and excellence strivers with high levels of goal attainment savored their positive sport experience and enjoyed their sport to a similar extent.

#### Complementary analyses

**Figure 7.**
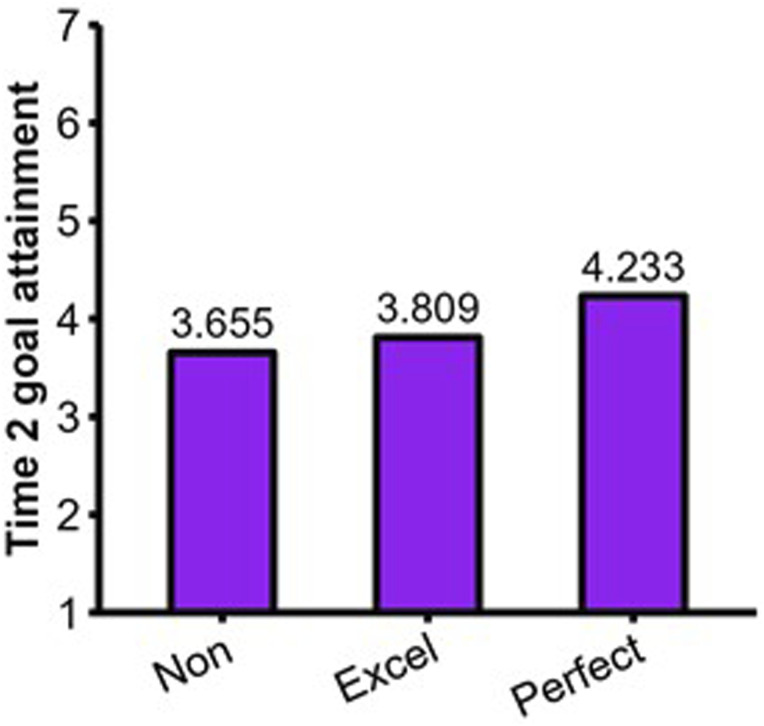
Results of complementary analyses for Time 2 goal attainment. Note. Non: Nonexcellence/nonperfection strivers; Excel: Excellence strivers; Perfect: Perfection strivers.

We performed a final multiple regression with excellencism and perfectionism as independent variables and Time 2 goal attainment as the dependent variable (Intercept = 3.94, *S.E.* = .097, *p* < .01). Results showed that excellencism was not a significant predictor (B = .098, *S.E.* = .118, *p* = .406, *β* = .073), whereas perfectionism was a significant positive predictor (B = .126, *S.E.* = .063, *p* = .047, *β* = .201). The predictors explained 5.9% of the variance in Time 2 goal attainment. The predicted values are displayed in Figure 7. Excellence strivers did not significantly differ from nonexcellence/nonperfection strivers (*d* = .146, 95% CI = [−.20, .49]). Perfection strivers had significantly higher goal attainment compared to both excellence strivers (*d* = .403, 95% CI = [.01, .80]) and nonexcellence/nonperfection strivers (*d* = .549, 95% CI = [.15, .95]). Interpretational scenario #4 of the MEP (see Figure 2) was the one that closely aligned with these findings.

### Brief discussion

Results both replicated and extended those of Study 1. Of particular interest, goal attainment moderated the associations between perfectionism and both savoring and sport enjoyment measured at Time 2. These findings can be used as derivatives to show that goal attainment moderated the differences between perfection strivers and excellence strivers on savoring (Figure 5) and sport enjoyment (Figure 6). At low and medium levels of goal attainment, perfection strivers have lower savoring and sport enjoyment than excellence strivers, thus supporting interpretational scenario #3 of the MEP (see Figure 2). At high levels of goal attainment, perfection and excellence strivers have comparable levels of savoring and sport enjoyment, thus supporting interpretational scenario #2 of the MEP (see Figure 2). Overall, we concluded that excellence strivers (compared to nonexcellence/nonperfection strivers) maintain higher savoring and sport enjoyment regardless of their levels of goal attainment; their sport experience is not contingent upon achievement. In contrast, perfection strivers (compared to excellence strivers) maintain comparably high savoring and sport enjoyment *only if they reach high levels of goal attainment*.

Our complementary analyses revealed that goal attainment was higher for the perfection strivers compared to excellence strivers. Perfectionism was beneficial for goal attainment in sport. This interpretation is defendable but recent studies have reported both negative (Waleriańczyk et al., 2023) and positive associations (Mallinson-Howard et al., 2021) with athletic performance. Prudence is therefore warranted. More convincingly, our findings indicate that perfectionism is a problem because the positive sport experience of perfection strivers is contingent on achievement. Once reinterpreted together, these findings indicate that perfectionism in sport can be seen as a double-edged sword (Stoeber, 2014) that entails better athletic achievement accompanied by important sacrifices in terms of savoring and enjoyment for athletes with lower perceived goal attainment.

## Discussion

The MEP has been described as “theory-driven research that moves the field ahead” (Smith et al., 2022, p. 48). In this study, our goal was to help the MEP fulfill its promises with a series of methodological-substantive steps designed to aid perfectionism researchers in their effort to incorporate excellencism in their research program. We developed a Shiny App and reported the results of two studies to underscore the usefulness of our approach. The nine interpretational scenarios of the MEP (see Figure 2) will lead the way to accelerate research while keeping it aligned with the founding principles of the theory. This study contributes to this agenda by clarifying how to use the effects of excellencism and perfectionism to estimate the predicted values of psychological outcomes associated with perfection strivers, excellence strivers, and nonexcellence/nonperfection strivers.

### Key findings

Other theoretical models, like the 2 × 2 (Gaudreau & Thompson, 2010) and tripartite models (e.g., Rice & Ashby, 2007), still focus on the difference between perfectionism and non-perfectionism to evaluate the adaptive and maladaptive outcomes of perfectionistic standards (for recent reviews, see Gaudreau, 2023; Gotwals & Lizmore, 2023). The current research reinforced the importance of being cautious about this comparison. In Study 1, we found that perfection strivers had higher savoring and were more likely to never experience dropout intention than nonexcellence/nonperfection strivers. In Study 2, similar findings were found for goal attainment as well as for savoring and sport enjoyment among athletes with medium and high levels of goal attainment.

Based on these findings, it would be tempting to conclude that perfectionism is adaptive. However, and more importantly, most of the effects of perfectionism are negative once we account for excellencism in the dualistic system advanced by the MEP. Excellencism is not just another variable thrown in the perfectionism landscape; it plays a central role required to fully understand the effects of perfectionism. Once reinterpreted together, these effects indicate that perfection strivers have lower savoring, sport enjoyment, and they are more likely to experience some dropout intentions than excellence strivers. Results of savoring—our primary dependent variable—replicated across distinct statistical analyses, all three temporal perspectives of savoring (past, present, future), and across cross-sectional and prospective/longitudinal designs. These findings meet the needed and sufficient conditions of the MEP to support the hypothesis that perfectionism is harmful (Gaudreau, 2019). Overall, the third interpretational scenario of the MEP (see Figure 2) was the one that most closely matched a majority of the results across our two studies.

One finding from Study 2 matched a pattern of effects that could point toward the relative benefits of perfectionism. Perfectionism (but not excellencism) was associated with significantly higher goal attainment in competitive athletes. Once reinterpreted together, these effects indicate that perfection strivers have higher predicted scores of goal attainment compared to excellence strivers. When considering goal attainment as a positive outcome, this finding supports the pattern of effects depicted in the fourth interpretational scenario of the MEP (see Figure 2).

Whether perfectionism is adaptive/healthy is an empirical question. The answer can vary across different dependent variables (e.g., performance and well-being) and populations (e.g., athletes and students). Showing that results can differ across dependent variables is not unexpected. As such, it illustrates the added value of the nine interpretational scenarios elaborated in the current research (see Figure 2). Without this framework, classifying and reconciliating contradictory results would remain difficult. Overall, the current research created a hub that will enable comparisons and integration of MEP findings in the years to come.

### A new look at moderation with the MEP

Detecting the presence of a moderating effect requires many thoughtful considerations. Lumping together two functionally distinct concepts (i.e., with different associations with outcomes) can create spurious effects (i.e., false positives) just as much as it can conceal (i.e., false negatives) the effect of theoretically defendable moderators. A clear distinction between perfectionism and excellencism is critical to re-envision the potential role of moderators.

In Study 2, goal attainment significantly moderated the associations of perfectionism with both savoring and sport enjoyment. Goal attainment did not significantly moderate the effects of excellencism. Once reinterpreted together, these effects indicate that the degree of difference between perfection and excellence strivers (in both savoring and sport enjoyment) varied across the amount of goal attainment reported by competitive athletes. Stated otherwise, different interpretational scenarios were at play at low (i.e., perfectionism is harmful; scenario 3) versus high levels of goal attainment (i.e., perfectionism is unneeded; scenario 2). The framework that we advanced in the current research provided a user-friendly roadmap to reinterpret conditional findings at different levels of a moderator.

Excellencism appears to relate to a more sustainable enduring capacity to savor and enjoy one’s sport. The better outcomes of excellence strivers (compared to nonexcellence/nonperfection strivers) were constant across low, medium, and high levels of goal. The positive sport experience of excellence strivers was not contingent upon their achievement. In contrast, perfection strivers and excellence strivers had comparable savoring and sport enjoyment only if they reached high level of goal attainment. Although the goal attainment of perfection strivers in sport was overall higher, their positive sport experience depended on their level of achievement. Perfection strivers espouse a winning at all cost mentality (Gaudreau & Schellenberg, 2022) that could explain their elevated goal attainment in sport. However, there seem to be a price to pay because they experience lower savoring and enjoyment in their sport at low and moderate levels of goal attainment. Perfectionism should be seen as tricky, particularly when people face goal-related adversity (i.e., low and moderate goal attainment). Perfection strivers experience a similar amount of savoring and enjoyment but only at high levels of goal attainment. This offers grounding for the idea that the positive psychological outcomes of those who pursue perfection are contingent on their capacity to attain their goals. In contrast, excellencism appears like a consistent adaptive resource across all levels of goal attainment.

### Required interpretational nuances

In this research, we advanced nine scenarios based on a 3 × 3 matrix of positive, null, and negative effects of excellencism and perfectionism (see Figure 2). This is a significant advance in the theory elaboration of the MEP. Our visual representation of these interpretational scenarios strictly focused on the valence of the effects (+, 0, −) without considering subtle variations in their strength. For most scenarios, changing the relative strength of the excellencism and perfectionism effects does not alter the fundamental interpretation. However, scenarios 3 and 7 can slightly differ depending on the relative strength of the excellencism and perfectionism effects (for another application of pattern effects in the perfectionism literature see Stoeber et al., 2020).

Let us take the results of our multiple regression (Study 1) to illustrate this point. Perfection strivers savored to a larger extent than nonexcellence/nonperfection strivers. In this case, the positive effect of excellencism was 2.91 times stronger than the negative effect of perfectionism (i.e., *B* = .370 vs. *B* = −.127). Keeping all other things constant, the difference between perfection strivers and nonexcellence/nonperfection strivers would become null if the positive effect of excellencism was 1.81 times stronger than the negative effect of perfectionism (i.e., *B* = .370 vs. *B* = −.204). In contrast, perfection strivers would savor to a smaller extent than nonexcellence/nonperfection strivers if the positive effect of excellencism would become 1.33 times stronger than the negative effect of perfectionism (i.e., *B* = .370 vs. *B* = −.278). The relative strength of the excellencism and perfectionism effects matters and this why it is important to visualize the predicted values of nonexcellence/nonperfection, excellence, and perfection strivers.

Research comparing excellence and perfection strivers will need to recruit sufficiently large samples. Attention will need to be paid to effect size and this is why the Cohen’s *d* values are a fundamental part of the interpretation of effects in the MEP. It would be important that effects comparable to the ones situated at the 50^th^ percentile in the personality literature (Bosco et al., 2015; Gignac & Szodorai, 2016) reach statistical significance in future research. The publication of null effects—estimated in sufficiently powered studies—will also be warranted to identify the life outcomes unaffected by personal standards (i.e., interpretational scenario 5) and the life outcomes unaffected by the difference between high standards and perfectionistic standards (interpretational scenarios 2, 5, and 8). Failure to reject the null hypothesis is insufficient to accept that an effect is null. Alternative approaches such as equivalence testing should be considered to evaluate if an effect can be seen as a negligible or practically zero (e.g., Alter & Counsell, 2021; Lakens et al., 2018). Gatekeeping to prevent the publication of null effects (by reviewers, editors, and researchers themselves) and the decision to selectively report the effects that fit one’s opinions and preferred scenario (i.e., cherry picking) should be avoided to paint a complete picture about the outcomes and processes that are affected and the ones that are unaffected by the pursuit of perfection over and above the pursuit of excellence.

### Limitations and future directions

In this research, we presented five statistical approaches to examine the excellencism and perfectionism effects. These statistical approaches will be appropriate across many research contexts and designs but other ones will require different analyses. In Study 2, we analyzed data collected from athletes at two points during their season. Such a prospective/longitudinal design is limited because it cannot be used to separate within-person and between-person effects (e.g., Orth et al., 2020). Measuring goal attainment, savoring, and enjoyment many times during the season (e.g., each month) would be required to clarify if perfectionism and excellencism lead to psychological adjustment or if the latter influences perfectionism and excellencism (e.g., Endleman et al., 2022; Negru-Subtirica et al., 2023).

Diaries and experiences sampling have been used to evaluate the daily (e.g., Dunkley, 2018) and weekly (e.g., Etherson et al., 2022) experiences of perfectionists. The framework presented in this study can easily be adapted to multilevel regression and multilevel structural equation modeling. Other researchers have examined the role of perfectionism in the development of performance and affective states (Curran & Hill, 2018; Gaudreau et al., 2019). Growth curve modeling with multilevel and structural equation modeling could follow the same steps as in our tutorial in order to compare the developmental trajectory (i.e., growth parameters) of excellence strivers and perfection strivers. Researchers could also examine the associations of excellencism and perfectionism with dependent variables that follow a multinomial distribution (e.g., different latent classes). The formulas used in the fourth example of this tutorial (i.e., the binary logistic regression available in the MEP Shiny App) could be extended to situations in which the dependent variable is categorized in more than two categories. It was impossible to cover all statistical analyses in this study but our approach offers the needed adaptability to be applicable across many variable-centered statistical analyses. Finally, we reanalyzed already published data and future meta-analyses should refrain from counting each of these reports as independent evidence.

### Conclusion

Our two studies contribute to an emerging line of research that calls attention to the essential role of excellencism to improve our understanding of perfectionism (Gaudreau et al., 2023). Excellencism and perfectionism are part of a dualistic system in which the former is a needed reference point to properly estimate the effects of perfectionism. The pivotal role of excellencism utterly affects all attempts to understand perfectionism. The mismeasurement of perfectionism (i.e., conflating high standards and perfectionistic standards into a single score) overestimates the positive outcomes and underestimates the negative outcomes of perfectionism. This measurement practice should stop in order to generate accurate effect size about the opportunity cost (ratio of pros and cons) of perfectionism. Clinical psychologists should be trained to recognize the subtle differences between excellencism and perfectionism in order to prioritize clients who are significantly more in need of urgent care. As evidence accumulates, the public discourse will evolve and everyone will be in a better position to determine when and for whom it could be appropriate to help people transform their perfectionism into excellencism.

Our interpretational scenarios and the MEP Shiny App are important additions to the toolkit of perfectionism researchers. With intuitive ease, our online calculator will help researchers to perform simulation (Gaudreau et al., 2021) or thought experiment (Jaccard & Jacoby, 2010) to formulate their hypotheses before their research, interpret their effects when reporting their results, and present the many possibilities of the MEP in their teaching and knowledge transfer activities. Not all researchers are using Mplus to perform their statistical analyses. Researchers using other software will be able to insert their results into the MEP Shiny App to calculate, compare, graph, and visualize the predicted values of nonexcellence/nonperfection, excellence, and perfection strivers and to calculate effect size. This will avoid manual calculations, minimize the risk of errors, and facilitate interpretation. The Shiny App will also help reviewers and editors to evaluate if authors interpret their findings in a way that is accurate with both the strength and the valence of the excellencism and perfectionism effects. In conclusion, this study not only deepens our understanding of perfectionism but also paves the way for making the MEP more accessible and practical for researchers and practitioners dealing with the intricate nature of perfectionism in their own work.

## Supplemental Material

Supplemental Material - From theory to research: Interpretational guidelines, statistical guidance, and a shiny app for the model of excellencism and perfectionism Supplemental Material for From theory to research: Interpretational guidelines, statistical guidance, and a shiny app for the model of excellencism and perfectionism by Patrick Gaudreau, Benjamin JI Schellenberg and Matthew Quesnel in European Journal of Personality

## Notes

1. Labels such as excellencist and perfectionist are not used to avoid equating people with their condition.

2. Null effects cannot be taken as evidence to support the null hypothesis in the null hypothesis significance testing paradigm; they indicate that support is insufficient/inconclusive for the positions that perfectionism is beneficial or harmful. Equivalent testing (see discussion) offers a promising avenue to evaluate the extent to which the statistically non-significant effects can be seen as sufficiently small to be considered negligible.

3. Outliers can distort the line of best fit and bias the parameter estimates. Sensitivity analyses were conducted without and with the outliers (see Table S1 and Table S2 of the supplementary file). Both in Study 1 and Study 2, parameter estimates and their statistical significance were unchanged with and without outliers.

4. Small differences on the third decimal point can be observed in the output from Mplus and the Shiny App because the former automatically uses the parameter estimates from the model without rounding.

5. The fit of the measurement and structural models are identical because all paths from the predictors to the dependent variable are freely estimated (i.e., equivalent models with the same number of degrees of freedom).

6. Two additional participants are excluded because they did not complete the dropout intention item.

## Appendix

### Predicted values (example 1–3)

B_0 =_ Intercept; B_1_ = excellencism; B_2_ = perfectionism; *SD* = standard deviation.

Nonexcellence/nonperfection strivers:

$$ŷ=B_{0}+\left(B_{1}\times \left(-SD{\;}_{\text{excel}}\right)\right)+\left(B_{2}\times \left(-SD{\;}_{\text{perfect}}\right)\right)$$

Excellence strivers:

$$ŷ=B_{0}+\left(B_{1}\times \left(SD{\;}_{\text{excel}}\right)\right)+\left(B_{2}\times \left(-\text{SD}{\;}_{\text{perfect}}\right)\right)$$

Perfection strivers:

$$ŷ=B_{0}+\left(B_{1}\times \left(SD{\;}_{\text{excel}}\right)\right)+\left(B_{2}\times \left(SD{\;}_{\text{perfect}}\right)\right)$$

### Predicted values (example 4)

Parameters of the count component of the model (frequency of dropout intention): B_0 =_ Intercept; B_1_ = excellencism; B_2_ = perfectionism; *SD* = standard deviation.

#### Predicted values for frequency of dropout intention

Nonexcellence/nonperfection strivers:

$$ŷ=\exp\left(B_{0}+\left(B_{1}\times \left(-SD{\;}_{\text{excel}}\right)\right)+\left(B_{2}\times \left(-SD{\;}_{\text{perfect}}\right)\right)\right)$$

Excellence strivers:

$$ŷ=\exp\left(B_{0}+\left(B_{1}\times SD{\;}_{\text{excel}}\right)+\left(B_{2}\times \left(-SD{\;}_{\text{perfect}}\right)\right)\right)$$

Perfection strivers:

$$ŷ=\exp\left(B_{0}+\left(B_{1}\times SD{\;}_{\text{excel}}\right)+\left(B_{2}\times SD{\;}_{\text{perfect}}\right)\right)$$

#### Predicted values for probability of zero dropout intention

Nonexcellence/nonperfection strivers:

$$ŷ=\exp\left(B_{0}+\left(B_{1}\times \left(-SD{\;}_{\text{excel}}\right)\right)+\left(B_{2}\times \left(-SD{\;}_{\text{perfect}}\right)\right)\right)/(\left(1\right)+\exp\;\left(B_{0}+\left(B_{1}\times \left(-SD{\;}_{\text{excel}}\right)\right)+\left(B_{2}\times \left(-SD{\;}_{\text{perfect}}\right)\right)\right)$$

Excellence strivers:

$$ŷ=\exp\left(B_{0}+\left(B_{1}\times SD{\;}_{\text{excel}}\right)+\left(B_{2}\times \left(-SD{\;}_{\text{perfect}}\right)\right)\right)/(\left(1\right)+\exp\;\left(B_{0}+\left(B_{1}\times SD{\;}_{\text{excel}}\right)+\left(B_{2}\times \left(-SD{\;}_{\text{perfect}}\right)\right)\right)$$

Perfection strivers:

$$ŷ=\exp\left(B_{0}+\left(B_{1}\times SD{\;}_{\text{excel}}\right)+\left(B_{2}\times SD{\;}_{\text{perfect}}\right)\right)/(\left(1\right)+\exp\;\left(B_{0}+\left(B_{1}\times SD{\;}_{\text{excel}}\right)+\left(B_{2}\times SD{\;}_{\text{perfect}}\right)\right)$$

Note. The same equations will be used in a binary logistic regression. For an example, see Gaudreau et al. (2022), Study 3; Straight A students vs. others).
