# Supplementary material for: From theory to research: Interpretational guidelines, statistical guidance, and a shiny app for the model of excellencism and perfectionism
Source: Eur J Pers. 2023 Dec 20;38(5):839–60. doi: 10.1177/08902070231221478 (PMC13021043; doi:10.1177/08902070231221478)
Supplement: Supplemental Material - From theory to research: Interpretational guidelines, statistical guidance, and a shiny app for the model of excellencism and perfectionism [file sj-pdf-1-erp-10.1177_08902070231221478.pdf]

SUPPLEMENTARY FILE

From Theory to Research: Interpretational Guidelines, Statistical Guidance, and a Shiny App for the Model of Excellencism and Perfectionism

Goal #1

Our first goal in this supplementary file was to present a summary table (see Table S1) to help navigate the syntax codes, outputs, figures of predicted values, and the Shiny App across the five analyses performed in our two studies. All annotated syntax codes and outputs are publicly available and anonymized for peer review: https://osf.io/8fb49

Goal #2

Our second goal was to perform sensitivity analyses to evaluate if the exclusion of outliers changed the results and interpretations of our findings from Study 1. In Study 1, we rerun our analyses after including the four outliers. As shown in Table S2, parameter estimates were very similar and interpretations remained unchanged with and without the outliers. In Study 2, we rerun our main analyses after including the two outliers. As shown in Table S3, parameter estimates were very similar and interpretations remained unchanged with and without the outliers. To stay consistent with our plan of analysis and previous MEP research, we conducted our final analyses without the outliers.

Table S1

Overview of the Analyses, Syntax Codes, Outputs, Figures, and R Studio Application (Shiny App)

Example	Model	Mplus syntax	Mplus output	Figures	Shiny App
# 1	Multiple regression	ex1_reg.inp	ex1_reg.out	Figure 3A	Regression-like models
		ex1_study2.inp	ex1_study2.out	Figure 7	
# 2	Multivariate multiple regression	ex2_multivreg.inp	ex2_multivreg.out	Figure 3B to 3D	Regression-like models
# 3	Structural equation modeling with latent variables	ex3_sem_measurement.inp	ex3_sem_measurement.out	----	----
		ex3_sem_structural.inp	ex3_sem_structural.out	Figure 3E	Regression-like models
# 4	Zero-inflated binomial regression	ex4_nbi.inp	ex4_nbi.out	Figure 4B	Binary logistic
				Figure 4C	Zero-inflated negative binomial
#5	Moderated multiple regression	ex5_modreg_vd1.inp	ex5_modreg_vd1.out	Figure 5	Regression-like models; enter the intercepts and slopes estimated separately at low, medium, and high values of the moderator
		ex5_modreg_vd2.inp	ex5_modreg_vd2.out	Figure 6	

Note. The Shiny App is available at: https://model-of-excellencism-and-perfectionism.shinyapps.io/Shiny_Version2/

Table S2

Study 1 Results of Sensitivity Analyses Without and With the Outliers Included in the Analyses

Analyses / Dependent variables	Excellencism				Perfectionism			
	B_0	B_1	*SE*	β_1	B_2	*SE*	β_2	R^2
**Multiple regression** [a]								
Savoring – composite score								
Without outliers	5.204	0.370	0.052	.412**	-0.127	0.033	-.254**	.131
With outliers	5.169	0.403	0.054	.436**	-0.128	0.035	-.247**	.139
**Multivariate multiple regression** [a]								
Anticipatory savoring								
Without outliers	5.196	0.437	0.062	.418**	-0.159	0.038	-.274**	.137
With outliers	5.165	0.465	0.064	.443**	-0.159	0.039	-.270**	.146
Momentary savoring								
Without outliers	5.037	0.280	0.060	.281**	-0.082	0.039	-.148*	.060
With outliers	4.998	0.320	0.063	.312**	-0.082	0.041	-.143*	.072
Reminiscence savoring								
Without outliers	5.379	0.394	0.058	.391**	-0.140	0.038	-.250**	.119
With outliers	5.345	0.423	0.059	.413**	-0.143	0.040	-.249**	.126
**Structural equation modeling** [a]								
Savoring – latent variable								
Without outliers	5.196	0.452	0.071	.461**	-0.164	0.041	-.304**	.167
With outliers	5.165	0.488	0.074	.485**	-0.164	0.042	-.298**	.174
**Zero-inflated negative binomial**								
Probability of zero dropout								
Without outliers, *N* =269	0.059	0.850	0.217	.395**	-0.300	0.101	-.252**	.122
With outliers, *N* =273	0.050	0.850	0.217	.406**	-0.286	0.214	-.244**	.122
Frequency of dropout								
Without outliers, *N* =269	0.771	-0.023	0.091	-.020	0.006	0.057	.009	n/a
With outliers, *N* =273	0.767	-0.016	0.090	-.015	0.010	0.056	.017	n/a

Note. [a] N = 271 (without outliers), N = 275 (with outliers). ** p <.01. * p < .05.

Table S3

Study 2 Results of Sensitivity Analyses Without and With the Outliers Included in the Main Analyses

	Time 2 Savoring			Time 2 Enjoyment		
	B	*SE*	*β*	B	*SE*	*β*
**Intercept**						
Without outliers	4.871	---	---	4.373	---	---
With outliers	4.870			4.372		
**Excellencism (E)**						
Without outliers	0.218*	0.091	.237*	0.373**	0.068	.436**
With outliers	0.209*	0.089	.235*	0.333**	0.065	.407**
**Perfectionism (P)**						
Without outliers	-0.118**	0.040	-.276**	-0.076*	0.030	-.190*
With outliers	-0.118**	0.040	-.278**	-0.078*	0.030	-.200*
**Goal attainment (GA)**						
Without outliers	0.070	0.054	.102	0.169**	0.052	.265**
With outliers	0.069	0.052	.104	0.160**	0.051	.260**
**E × GA**						
Without outliers	0.050	0.083	.051	-0.130	0.082	-.142
With outliers	0.064	0.076	.075	-0.071	0.078	-.090
**P × GA**						
Without outliers	0.062*	0.028	.166*	0.054*	0.027	.157*
With outliers	0.062*	0.026	.180*	0.049†	0.026	.155*

Note. [a] *N* = 296 (without outliers), *N* = 298 (with outliers). ** p <.01. * p < .05. † p = .053.

Goal #3

We used the results of total savoring (Study 1, example #1) to provide an example to illustrate the possible misinterpretations of error bars when trying to visually display the differences across nonexcellence/nonperfection, excellence, and perfection strivers. Researchers often use 95% CI of the predicted values to illustrate the dispersion around point estimates. Things get complicated when people try to interpret if two points are significantly different using the error bars. In our example, the 95% CI intervals of savoring for perfection strivers overlapped with the 95% CI intervals of savoring for nonexcellence/nonperfection strivers (see Figure S1, Panel A). Based on the overlap, it would be tempting to conclude that these two predicted values do not significantly differ from one another. This is a classic interpretation mistake previously discussed in the literature, but still largely unknown in psychological sciences (Knol et al., 2011; Schenker & Gentleman, 2001).

As shown elsewhere, overlapping 95% CIs cannot be taken as evidence for a non-significant difference between estimates (Cumming & Finch, 2005; Schenker & Gentleman, 2001). Examining the overlap is a conservative approach (Schenker & Gentleman, 2001) because non-overlapping 95% CIs correspond to a p value smaller than .01 (Knol et al., 2011). In our example, the exact p value associated with our standardized difference between perfection strivers and nonexcellence/nonperfection strivers (Cohen's $d = 0.316$, $p = .016$) showed that the difference was statistically significant. Furthermore, the 95% CI of Cohen's d did not include zero (95% CI = [0.059, 0.573]), which also indicated that the difference between perfection strivers and nonexcellence/nonperfection strivers was statistically significant. Prudence is therefore warranted when using error bars to compare predicted values of

nonexcellence/nonperfection, perfection, and excellence strivers. Inferential tests should have precedence to avoid interpretation errors.

Researchers have suggested using an adjusted 95% CI to align the associated probability more closely with the outcome of conventional significance testing (Cousineau, 2017)[1]. Instead of using 95% CIs, the adjusted approach uses 83.4% confidence intervals (see Formula 10 in the supplementary material of Knol et al., 2011)[2]. At first glance, it is tempting to use the overlap of adjusted 95% CIs as a rule-of-thumb to assess whether there is a significant difference ($p < .05$) between two values. However, as shown in panel B of Figure S1, overlap between adjusted confidence intervals can still occur even if there is a significant difference between two predicted values. This is a known problem: "Rejection of the null hypothesis by the method of examining overlap implies rejection by the standard method, whereas failure to reject by the method of examining overlap does not imply failure to reject by the standard method" (Schenker & Gentleman, 2001, p. 182). The adjusted 95% CI of the predicted values (and the corresponding error bars in the figure) are provided as an option in our Shiny App. However, we strongly discourage making interpretational decisions based on visual inspection of adjusted confidence intervals. "Although the method of examining overlap is simple and especially convenient when lists or graphs of confidence intervals have been presented, we conclude that it should not be used for formal significance testing" (Schenker & Gentleman, 2001, p. 182). We decided to present figures without error bars to ensure that our results are accurately understood and cited by readers.

Figure S1

[1] This approach appears to be implemented in Mplus. The 95% of the predicted values in Mplus and our MEP Shiny App are not identical but highly similar (potentially due to rounding issues).
[2] The standard error is multiplied by a value of 1.3859 (Knol et al., 2011) rather than 1.96 to obtain 83.4% confidence intervals. This approach is used in our MEP Shiny App.

Predicted Values with their 95% Confidence Intervals (Panel A) and 95% Adjusted Confidence Intervals (Panel B)

Panel A

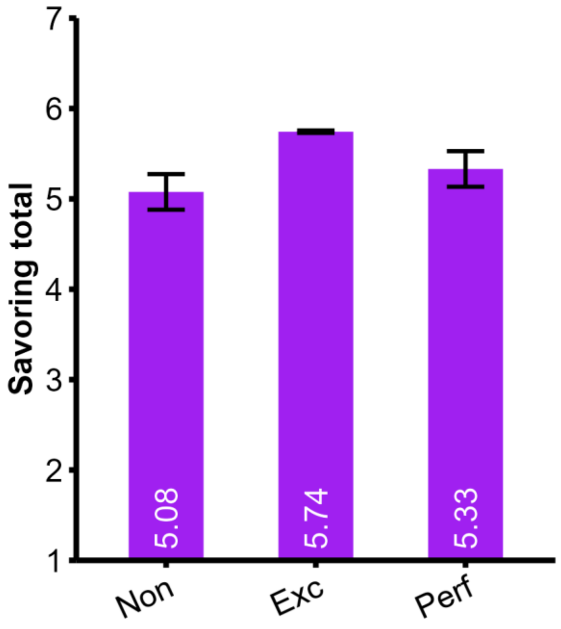

Panel B

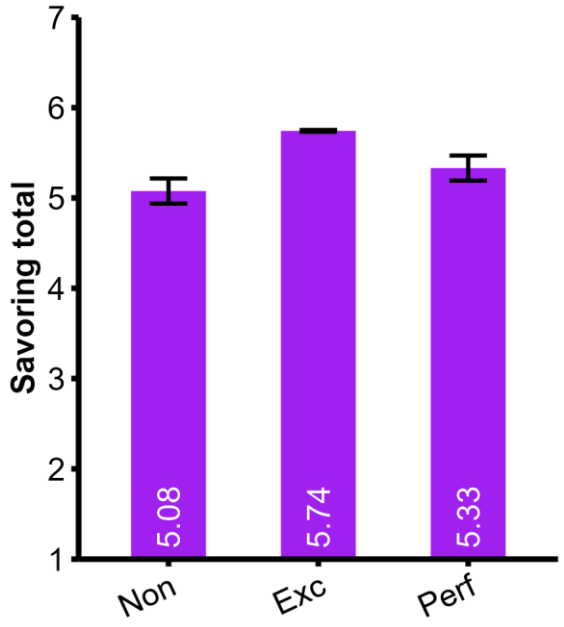

The 95% CI (Panel A) and the adjusted 95% CI (Panel B) of perfection strivers and nonexcellence/nonperfection strivers are overlapping. However, the standardized difference is statistically significant (Cohen's *d* = 0.316, *S.E.* = 0.131, *p* = .016). The 95% CI of this standardized difference (95% CI = [0.059, 0.573]) does not contain zero. The two predicted values significantly differ ($p < .05$) even if their 95% CI are overlapping. Our example shows that "although the method of examining overlap is simple and especially convenient when lists or graphs of confidence intervals have been presented, we conclude that it should not be used for formal significance testing" (Schenker & Gentleman, 2001, p. 182).

References in the supplementary file (all included in the manuscript)

Cousineau, D. (2017). Varieties of confidence intervals. *Advances in Cognitive Psychology*, *13*(2), 140-155. https://doi.org/10.5709/acp-0214-z

Cumming, G., & Finch, S. (2005). Inference by eye: Confidence intervals and how to read pictures of data. *American Psychologist*, *60*(2), 170-180. https://doi.org/10.1037/0003-066x.60.2.170

Knol, M. J., Pestman, W. R., & Grobbee, D. E. (2011). The (mis)use of overlap of confidence intervals to assess effect modification. *European Journal of Epidemiology*, *26*(4), 253-254. https://doi.org/10.1007/s10654-011-9563-8

Schenker, N., & Gentleman, J. F. (2001). On judging the significance of differences by examining the overlap between confidence intervals. *The American Statistician*, *55*(3), 182-186. https://doi.org/10.1198/000313001317097960
